# Analysis of immunogenic cell death in ascending thoracic aortic aneurysms based on single-cell sequencing data

**DOI:** 10.3389/fimmu.2023.1087978

**Published:** 2023-05-03

**Authors:** Zemin Tian, Peng Zhang, Xinyang Li, Delong Jiang

**Affiliations:** ^1^ Department of Vascular and Thyroid Surgery, The First Affiliated Hospital of China Medical University, Shenyang, Liaoning, China; ^2^ Department of Neurology, The First Affiliated Hospital of Kunming Medical University, Kunming, Yunnan, China

**Keywords:** ICD (immunogenic cell death), ATAA (ascending thoracic aortic aneurysms), ACKR1, CXCL12 (SDF-1α), CTL (cytotoxic T cells)

## Abstract

**Background:**

At present, research on immunogenic cell death (ICD) is mainly associated with cancer therapy. Little is known about the role of ICD in cardiovascular disease, especially in ascending thoracic aortic aneurysms (ATAA).

**Method:**

ATAA single-cell RNA (scRNA) sequencing data were analyzed to identify the involved cell types and determine their transcriptomic characteristics. The chi-square test, Gene Ontology (GO) and Kyoto Encyclopedia of Genes and Genomes (KEGG) enrichment analyses, Gene Set Enrichment Analysis (GSEA), and CellChat for cell-to-cell communication analysis from the Gene Expression Omnibus (GEO) database were used.

**Result:**

A total of 10 cell types were identified, namely, monocytes, macrophages, CD4 T/NK (CD4+ T cells and natural killer T cells), mast cells, B/Plasma B cells, fibroblasts, endothelial cells, cytotoxic T cells (CD8+ T cells, CTLs), vascular smooth muscle cells (vSMCs), and mature dendritic cells (mDCs). A large number of inflammation-related pathways were present in the GSEA results. A large number of ICD-related pathways were found in the KEGG enrichment analysis of differentially expressed genes in endothelial cells. The number of mDCs and CTLs in the ATAA group was significantly different from that in the control group. A total of 44 pathway networks were obtained, of which 9 were associated with ICD in endothelial cells (CCL, CXCL, ANNEXIN, CD40, IL1, IL6, TNF, IFN-II, GALECTIN). The most important ligand−receptor pair by which endothelial cells act on CD4 T/NK cells, CTLs and mDCs is CXCL12-CXCR4. The most important ligand−receptor pair by which endothelial cells act on monocytes and macrophages is ANXA1-FPR1. The most important ligand−receptor pair by which CD4 T/NK cells and CTLs act on endothelial cells is CCL5-ACKR1. The most important ligand−receptor pair that myeloid cells (macrophages, monocytes and mDCs) act on endothelial cells is CXCL8-ACKR1. Moreover, vSMCs and fibroblasts mainly promote inflammatory responses through the MIF signaling pathway.

**Conclusion:**

ICD is present in ATAA and plays an important role in the development of ATAA. The target cells of ICD may be mainly endothelial cells, in which the aortic endothelial cell ACKR1 receptor can not only promote T-cell infiltration through the CCL5 ligand but also promote myeloid cell infiltration through the CXCL8 ligand. ACKR1 and CXCL12 may become target genes for ATAA drug therapy in the future.

## 1 Introduction

It is well known that ascending thoracic aortic aneurysms (ATAA) are asymptomatic until complications such as rupture and dissection are present (1–3). The adventitia and media of the thoracic aortic wall contain high levels of inflammatory cells. In particular, studies have demonstrated that macrophages and T lymphocytes are prevalent in the thoracic aortas of patients with sporadic ATAA (4–6). Apoptosis, a type of regulatory cell death, has been shown to be significantly increased in smooth muscle cells (SMCs) in ATAA (7, 8). This observation also implies that there is regulated cell death in ATAA, and we explore the role of immunogenic cell death (ICD) in ascending thoracic aortic aneurysms in this study.

ICD, a type of regulated cell death, can lead to an inflammatory response, which triggers cytotoxic T lymphocyte (CTL)-driven adaptive immunity, as well as long-term immunological memory (9). It is well known that ICD occurs mainly in three types of cells: dying cells, antigen-presenting cells (APCs), and cytotoxic T cells. Moreover, three conditions need to be met for ICD to occur: antigenicity, adjuvanticity, and favorable microenvironment (10).

In this study, we first demonstrated the existence of ICD in ATAA. Second, we identified that the target cells for ICD were mainly endothelial cells of the ascending aorta. Finally, we identified the most important ligands released by endothelial cells and the most important receptors expressed by endothelial cells that cause ICD. In conclusion, investigating ICD in ATAA may provide new ideas for ATAA targeted therapy.

## 2 Methods

### 2.1 scRNA sequencing data processing

ATAA cells were collected by flow cytometry sorting and submitted to a 10X Chromiun System with an Illumina NovaSeq 6000 (1). To generate the barcode, gene, and expression matrix files, the cleaned data (GSE155468) were aligned to the human reference genome (GRCh38 transcriptome) using Cell Ranger (version 3.0.2, 10X Genomics). In the GSE155468 dataset, there were samples from 11 individuals, including 8 samples in the experimental ATAA group and 3 samples in the control group (The protocol for collecting human tissue samples was approved by the Institutional Review Board at Bayer College of Medicine (1)). In the Seurat R package, we first used PCA to reduce the dimensionality of the downstream data and then used t-distributed stochastic neighbor embedding (t-SNE) analysis to reduce the dimensionality of the data again (11). We removed cells with fewer than 200 genes, more than 7,000 genes, and more than 10% mitochondrial genes. Analysis was performed on 48128 filtered cells. Subsequent analysis was performed on 48128 filtered cells. Using the “LogNormalize” function, gene expression was normalized and scaled. Each sample possessed 2000 highly variable genes (HVGs) using the vst method after data normalization. After identifying significant principal components (PCs), PCA was applied. Batch correction was performed using the “Harmony” R package (version 0.1.0) (12) to avoid batch effects resulting from sample identity that could disrupt downstream analysis. Finally, 50 PCs were selected for t-SNE analysis. A total of 60 distinct clusters were created using FindClusters function at 4.0 resolution, and these clusters were then grouped into 10 cell types using marker genes, and the results of the “FindAllMarkers” function were manually checked for match with marker gene results. In each cluster, differentially expressed genes (DEGs) were identified using the “FindAllMarkers” function with logfc.threshold = 0.25 (13). Thirty-four ICD marker genes were selected, and a heatmap was constructed using the 10 identified cell types (14).

### 2.2 Gene ontology (GO) and Kyoto encyclopedia of genes and genomes (KEGG) analyses and gene set enrichment analysis (GSEA)

Significantly differentially expressed genes in each cell group relative to the other groups were identified and subjected to GSEA. The GO and KEGG enrichment analysis of the obtained smooth muscle cell differentially expressed genes and the obtained endothelial cell differentially expressed genes were performed using the GSE155468 dataset (logfc.threshold = 0, P_val_adj<0.05), and the GO and KEGG enrichment results of the differentially expressed genes between the two cell populations were compared (15).

### 2.3 Statistical analysis

Differences between the ATAA group and control group were evaluated with a chi square (χ²) test or Fisher exact test for all categoric variables. Data were analyzed using IBM SPSS Statistics 2 software (version 26.0; IBM Corp, Armonk, NY, USA).

### 2.4 Cellchat in ATAA

First, in the ATAA data, we used the CellChat and patchwork packages to create CellChat objects, set up the ligand−receptor interaction database, and preprocessed the expression data for cell communication analysis.

Second, we used CellChat to infer biologically meaningful communication, and used the “trimean” function to calculate communication probability and infer the CellChat network. Then, we extracted the inferred CellChat network as a data frame; used the signaling pathway level to infer communication; and computationally integrated cellular communication networks (**Supplementary Figure 1**).

Third, we used circle plots to visualize signal paths that may be related to ICDs (**Supplementary Figures 2**–**10**). We calculated the contribution of each ligand-receptor pair to these signaling pathways, and visualized cellular communication regulated by individual ligand-receptor pairs. Signal gene expression distributions were plotted using violin plots for these communications.

**Figure 1:**
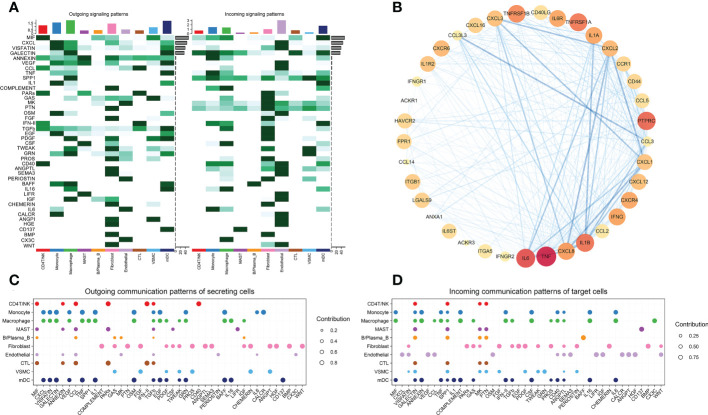
**(A)** Identified signals that contribute the most to the outgoing or incoming signaling of individual cell groups. **(B)** PPI analysis of ICD signaling pathway genes. **(C)** Outgoing communication patterns of secreting cells. **(D)** Incoming communication patterns of target cells.

Fourth, a systematic analysis of cellular communication networks was performed to identify the signaling roles of cell groups (e.g., dominant transmitter, receiver) as well as the main contributing signals. We identified the signals that contributed the most to the efferent or afferent signaling of certain cell groups (**Figures 1A, C, D**).

**Figure 2:**
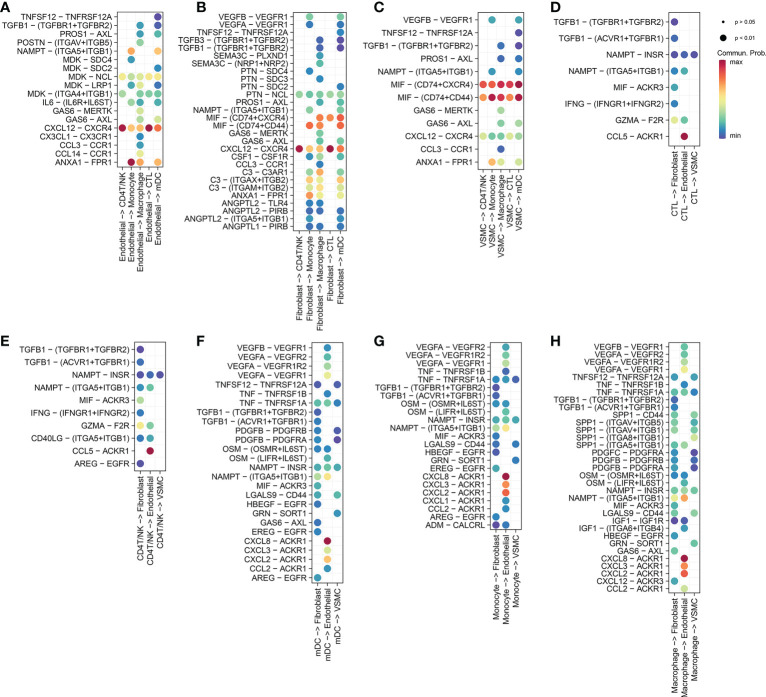
**(A)** Endothelial cells release ligands that act on myeloid cells (macrophages, monocytes and dendritic cells) and T cells (CD4 T/NK cells and cytotoxic T cells). **(B)** Fibroblasts release ligands that act on myeloid cells and T cells. **(C)** VSMCs release ligands that act on myeloid cells and T cells. **(D)** Cytotoxic T cells release ligands that act on nonimmune cells (fibroblasts, VSMCs, endothelial cells). **(E)** CD4 T/NK cells release ligands that act on nonimmune cells. **(F)** Mature dendritic cells release ligands that act on nonimmune cells. **(G)** Monocytes release ligands that act on nonimmune cells. **(H)** Macrophages release ligands that act on nonimmune cells.

We compared the ICD-related pathways in which endothelial cells act as ligands on other cells, and identified the most important pathways. We used the same method to compare macrophages, monocytes, mDCs, CD4 T/NK cells and cytotoxic T cells (**Figure 2**). Protein−protein interaction (PPI) networks were constructed using the most important ligand and receptor genes and visualized using Cytoscape software (**Figure 1B**).

## 3 Results

### 3.1 ATAA scRNA profiling

**Figure 3:**
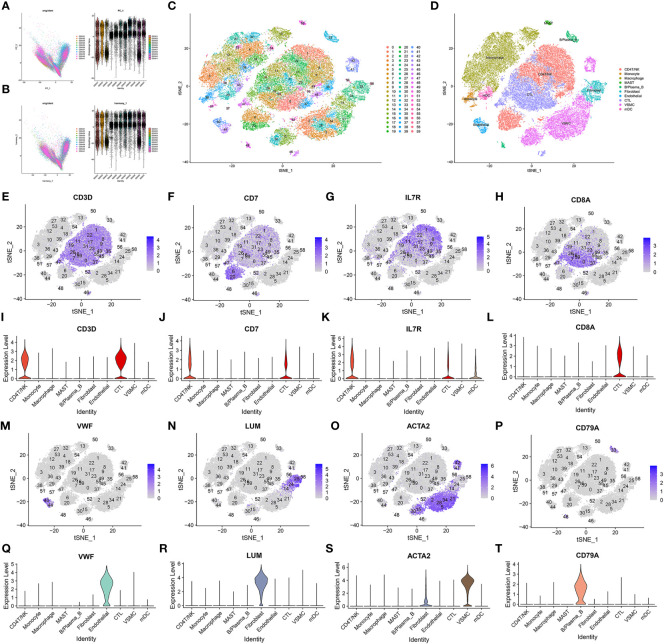
Data processing and defining cell types. **(A, B)** Harmony package sample batch effect elimination and PCA dimensionality reduction. **(C)** t-SNE dimensionality reduction. **(D)** The t-SNE results were divided into 10 cell populations using marker genes. **(E–T)** Sixty clusters defined using marker genes.

After filtering the data, there were a total of 48,128 cells in the scRNA sequencing dataset (GSE155468), including 39,651 cells from patients with ATAA and 8,477 cells from control individuals (**Tables 1**–**5**). After PCA, and Harmony, and t-SNE processing of the dataset (**Figures 3A, B**), we examined results (**Figure 3C**).

**Table 1:** Demographic characteristics of patients and controls (1).

Variable	ATAA1	ATAA2	ATAA3	ATAA4	ATAA5	ATAA6	ATAA7	ATAA8	Control4	Control6	Control9
**Sex**	F	F	M	M	F	F	M	M	F	M	F
**Ethnicity**	Non-Hispanic	Non-Hispanic	Non-Hispanic	Non-Hispanic	Non-Hispanic	Non-Hispanic	Non-Hispanic	Non-Hispanic	Non-Hispanic	Non-Hispanic	Hispanic
**Race**	White	White	White	White	White	White	White	White	White	Black	Latino
**Age (y)**	75	78	59	62	75	67	69	56	63	61	62
**Diagnosis/ Comments**	ATAA	ATAA	ATAA with root aneurysm	ATAA	ATAA with root aneurysm	ATAA with arch and DTAA	ATAA with root aneurysm	ATAA with root aneurysm	Heart transplant recipient	Heart transplant recipient	Lung transplant donor
**Aortic diameter (cm)**	5.2	4.9	5	5.2	5.8	4.9	5.2	5.2	NA	NA	2.2
**Smoking status**	Past (quit before 1990)	Never	Never	Never	Past (quit 1999)	Never	Never	Never	Never	Past	Current
**Diabetes**	No	No	Yes	Yes	No	No	No	No	No	No	Yes
**Hypertension**	Yes	Yes	Yes	Yes	Yes	Yes	Yes	Yes	No	Yes	Yes
**COPD**	No	Yes	No	No	Yes	No	No	No	No	No	No
**Aortic valve regurgitation**	No	No	Yes	Yes	Yes	Yes	No	Yes	No	No	No
**BAV**	No	Yes	Yes	No	No	No	NA	No	NA	NA	No
**Re-operation**	No	No	Yes *	No	No	No	Yes **	No	No	No	No

*Previous arch debranching before stent graft repair of arch and descending thoracic aorta. **Previous aortic valve replacement. NA, not available.

**Table 2:** Dataset features.

Datasets	Type	Platform	Sample size (Control/ATAA)
**GSE155468**	scRNA sequencing	Illumina NovaSeq 6000 (Homo sapiens)	3,8

**Table 3:** Single sample data features.

Sample name	Raw data	Data filtering (200<nFeature-RNA<7000,percent.mt<10)
**GSM31 (Control 4: 63 years)**	3377	3377
**GSM32 (Control 6: 61 years)**	1193	1193
**GSM33 (Control 9: 62 years)**	3907	3907
**GSM34 (ATAA 1: 75 years)**	6686	6686
**GSM35 (ATAA 2: 78 years)**	4598	4598
**GSM36 (ATAA 3: 59 years)**	3622	3622
**GSM37 (ATAA 4: 62 years)**	6384	6384
**GSM38 (ATAA 5: 75 years)**	4802	4802
**GSM39 (ATAA 6: 67 years)**	3526	3526
**GSM40 (ATAA 7: 69 years)**	6997	6997
**GSM41 (ATAA 8: 56 years)**	3036	3036
**All (ATAA: 67.6±8.1 years, Control: 62±1 years)**	48128	48128

**Table 4:** Data characteristics of the control group.

cell types	number of cells	ratio(%)
CD4T/NK	582	6.87
Monocyte	77	0.91
Macrophage	1917	22.61
MAST	61	0.72
B/Plasma_B	57	0.67
Fibroblast	319	3.76
Endothelial	364	4.29
CTL	338	3.99
VSMC	4674	55.14
mDC	88	1.04
All	8477	100

**Table 5:** Data characteristics of the ATAA group.

cell types	number of cells	ratio (%)
**CD4T/NK**	11852	29.89
**Monocyte**	725	1.83
**Macrophage**	9724	24.52
**MAST**	207	0.52
**B/Plasma_B**	859	2.17
**Fibroblast**	1557	3.93
**Endothelial**	565	1.42
**CTL**	8754	22.08
**VSMC**	4719	11.9
**mDC**	689	1.74
**All**	39651	100

**Figure 4:**
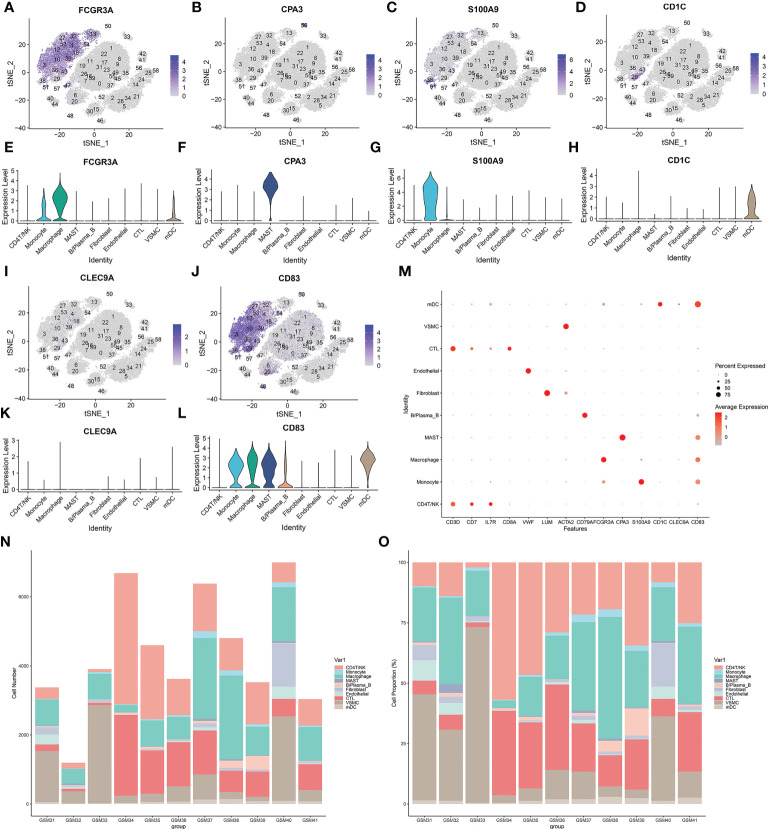
**(A–L)** Sixty clusters defined using marker genes. **(M)** The expression of cell type marker genes is shown in the dot plot. **(N–O)** Proportion of various types of cells in each sample.

Sixty clusters could be assigned to known cell lineages through marker genes (**Supplementary List 1**), according to a previous study. We used t-SNE analysis to visualize the 10 clusters (**Figure 3D**) and identify marker genes in the 10 cell-type populations (**Supplementary List 2**). The expression of cell type marker genes is shown in the dot plot (**Figure 4M**). We observed 10 cell clusters (CD4T/NK: clusters 1, 6, 8, 9, 11, 17, 20, 22, 31, 35, 45, 46 and 52; expressing CD3D (16) (**Figures 3E, I**), CD7 (17) (**Figures 3F, J**), IL7R (interleukin 7 receptor) (18) (**Figures 3G, K**); CTL: clusters 0, 7, 16, 19, 23, 26, 37, 47, 49, 55 and 59; expressing CD8A (19) (**Figures 3H, L**); Endothelial: clusters 40 and 44; expressing vWF (20) (von Willebrand factor) (**Figures 3M, Q**); Fibroblast: clusters 24, 25, 56 and 58; expressing LUM (Lumican) (21) (**Figures 3N, R**); vSMC: clusters 2, 5, 14, 15, 21, 28, 30, 34, 41 and 42; expressing ACTA2 (actin alpha 2, smooth muscle) (22) (**Figures 3O, S**); B/Plasma_B: clusters 33 and 48; expressing CD79A (23) (**Figures 3P, T**); Macrophage: clusters 3, 4, 10, 12, 13, 18, 27, 32, 36, 39, 43, 53 and 54; expressing FCGR3A (24) (**Figures 4A, E**); Mast: cluster 50; expressing CPA3 (25) (**Figures 4B, F**); Monocytes: clusters 38 and 51, expressing S100A9 (26) (**Figures 4C, G**); mDCs: clusters 29 and 57; expressing CD1C (27) (**Figures 4D, H**), CLEC9A (28) (**Figures 4I, K**), CD83 (29) (**Figures 4J, L**).

According to the results in **Table 4** and **Table 5**, T-cells accounted for 51.97% of all ATAA cells (**Figures 4N, O**). This finding suggests that T-cell infiltration plays a very important role in the pathogenesis of ATAA. In ATAA, the number of endothelial cells was significantly reduced, accounting for only 1.42%.

### 3.2 The results of GO and KEGG analyses and GSEA

**Figure 5:**
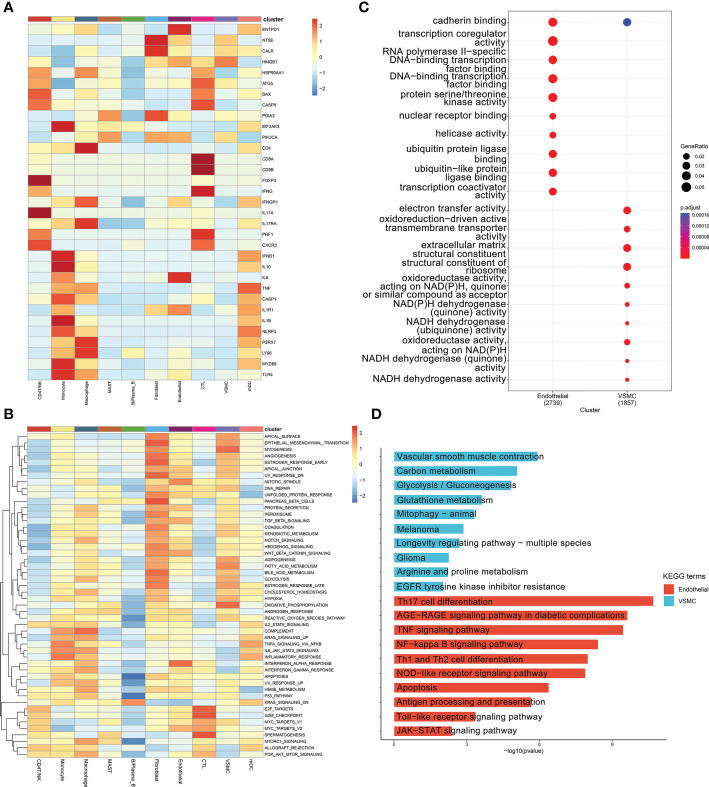
**(A)** ICD marker gene heatmap in ten cell types. **(B)** GSEA for differentially expressed genes between different cell types. **(C)** GO analysis of the differentially expressed genes between the endothelial and VSMC clusters. **(D)** KEGG analysis of the differentially expressed genes between the endothelial and VSMC clusters.

In nonimmune cells, 34 ICD-related marker genes were mainly expressed in endothelial cells (**Figure 5A**). In **Figure 5B**, we only showed the top 50 most important pathways, which could be mainly divided into several categories, such as metabolism-related, oxidative respiration-related, inflammation-related, and apoptosis-related, etc. At present, it is well known that the occurrence of cardiovascular disease was closely related to the abnormal expression of these pathways. Inflammation plays an important role in the occurrence and development of ATAA. When we compared the GO enrichment results of endothelial cells and VSMCs, we found that the pathways enriched in endothelial cells were mainly related to transcription, while the pathways enriched in VSMCs were mainly related to the oxidative respiratory chain (**Figure 5C**). When we compared the KEGG results, we found that differentially expressed genes in endothelial cells were enriched in a large number of ICD-related pathways (**Figure 5D**; **Supplementary List 3**).

### 3.3 Chi-square test results of the number of mDCs and cytotoxic T cells

The number of mature dendritic cells was significantly correlated with the formation of ATAA, K² (mDC) =136.86, P(K²>10.828) <0.001 (**Table 6**). The number of cytotoxic T cells was significantly correlated with the formation of ATAA, K²(CTL)=1601.41, P(K²>10.828) <0.001 (**Table 7**).

**Table 6:** mDC Chi-square test.

	mDCs	other cells	all cells
**ATAA**	**689**	38962	39651
**Normal**	**88**	8389	8477
	**777**	47351	48128

K²=136.86.

P(K²>10.828)<0.001.

Conclusion: The number of mDCs is associated with the formation of ATAA.

**Table 7:** CTL Chi-square test.

	CTL	other cells	
**ATAA**	8754	30897	39651
**Normal**	338	8139	8477
	9092	39036	48128

K²=1601.41.

P(K²>10.828)<0.001.

Conclusion:The number of CTL is associated with the formation of ATAA.

### 3.4 The ATAA CellChat results

We obtained a total of 44 cell-to-cell communications. Among nonimmune cells, the cells that sent the most cellular signals were fibroblasts, while the ones that received the most signals were endothelial cells (**Figure 1A**). This finding suggests that although VSMCs and fibroblasts may also trigger ICD through the MIF signaling pathway (**Figures 2B**, **C**), endothelial cells may be the main cells responsible for ICD. Therefore, we mainly explored ICD in endothelial cells. The 9 pathway networks are ANNEXIN signaling pathway network, CXCL signaling pathway network, CCL signaling pathway network, IFN-II signaling pathway network, IL1 signaling pathway network, IL6 signaling pathway network, GALECTIN signaling pathway network, TNF signaling pathway network, and CD40 signaling pathway network. Cells associated with these pathways were mainly endothelial cells, myeloid cells, CD4 T/NK cells and cytotoxic T cells (**Figures 1C, D**).

Endothelial cells acted as ligands in the ICD-related pathways of myeloid cells, including the ANNEXIN signaling pathway network (**Supplementary Figure 2**), CXCL signaling pathway network (**Supplementary Figure 3**), CCL signaling pathway network (**Supplementary Figure 4**) and IL6 signaling pathway network (**Supplementary Figure 10**).

In the ANNEXIN signaling pathway network, there was only one ligand-receptor pair, ANXA1-FPR1 pathway (**Supplementary Figure 2A**), and ANXA1 ligands were highly expressed in all cells except B/Plasma_B cells (**Supplementary Figure 2E**). The receptor FPR1 was only expressed in myeloid cells (**Supplementary Figure 2E**). Among all the ICD-related pathways in which endothelial cells acted as ligands for myeloid cells (macrophage, monocyte and mDC), ANXA1-FPR1 was the most contributing pair (**Figure 2A**).

In the CXCL signaling pathway network, a total of seven ligand-receptor pairs were obtained, namely CXCL8-ACKR1, CXCL12-CXCR4, CXCL2-ACKR1, CXCL3-ACKR1, CXCL16-CXCR6, CXCL12-ACKR3, and CXCL1-ACKR1 (**Supplementary Figure 3A**). Among them, CXCL8-ACKR1 contributed the most to the CXCL signaling pathway network. The CXCL8 ligand was highly expressed in myeloid cells, while the ACKR1 receptor was only expressed in endothelial cells (**Supplementary Figure 3K**). Among all the ICD-related pathways in which myeloid cells (macrophages, monocytes and mDC) acted as ligands to endothelial cells, CXCL8-ACKR1 was the most contributing pair (**Figures 2F, G, H**). Among all the ICD-related pathways in which endothelial cells and fibroblasts acted as ligands to T cells (CD4T/NK cells and cytotoxic T cells), CXCL12-CXCR4 was the most contributing pair (**Figures 2A, B**).

In the CCL signaling pathway network, we obtained a total of 7 ligand-receptor pairs, namely CCL5-ACKR1, CCL2-ACKR1, CCL3-CCR1, CCL14-ACKR1, CCL3L1-CCR1, CCL5-CCR1, and CCL14-CCR1 (**Supplementary Figure 4A**). Among these pairs, CCL5-ACKR1 contributed the most to the CCL signaling pathway network. As a ligand, CCL5 was only highly expressed in CD4T/NK cells and cytotoxic T cells, while the ACKR1 receptor was only expressed in endothelial cells (**Supplementary Figure 4K**). Among all the ICD-related pathways in which T cells (CD4T/NK cells and cytotoxic T cells) acted as ligands to endothelial cells, CCL5-ACKR1 was the most contributing pair (**Figures 2D, E**).

Myeloid cells (macrophage, monocyte and mDC) acted as ligands on the ICD-related pathways of T cells (CD4T/NK and CTL), including the CXCL signaling pathway network (CXCL16-CXCR6) (**Supplementary Figure 3**), TNF signaling pathway network (TNF-TNFRSF1B) (**Supplementary Figure 6**), and GALECTIN signaling pathway network (LGALS9-CD44 and LGALS9-CD45) (**Supplementary Figure 5**; **Figures 5B, D, E**). Among them, the contribution of the LGALS9-CD45 (PTPRC) pathway was the largest.

## 4 Discussion

ICD has not been reported in ATAA before, and almost all recent studies have mainly focused on ICD in the context of cancer therapy (30–32). In our study, we found that the marker genes of ICD were highly expressed in endothelial cells, and KEGG enrichment analysis results showed that the differentially expressed genes were enriched in a large number of ICD-related pathways. Based on the cell-to-cell communication results, we found that endothelial cells received the most signals and that there were numerous signaling pathways associated with ICD. This result suggests that dying endothelial cells in ATAA may contribute to the progression of ATAA through ICD. We identified the most important ligand−receptor pair leading to endothelial cell ICD, and according to these results, found that endothelial cells mainly acted on APCs and T cells by releasing ANXA1 and CXCL12 chemokines, respectively. The chemokine CXCL8 released by APCs and the chemokine CCL5 released by T cells bind to ACKR1 expressed by endothelial cells.

It is well known that ICD requires the simultaneous satisfaction of three conditions: antigenicity, adjuvanticity, and microenvironment (10, 33). First, we explored antigenicity in ATAA. In the field of oncology, it is currently believed that dying tumor cells can provide antigenicity by conventional methods of generating epitopes (10). However, in nontumor cells, cellular oxidation could also cause enzymatic or nonenzymatic posttranslational modifications (PTMs) that generate epitopes that initiate ICD (34). In cardiovascular disease, endothelial cell dysfunction caused by oxidative stress stimulates abnormal proinflammatory and prothrombotic phenotypes of the endothelial cells lining the lumen of blood vessels (35, 36). This observation partly suggests that dying endothelial cells in ATAA can indeed generate epitopes that initiate ICD. The proinflammatory capacity of dysfunctional endothelial cells also provides a favorable microenvironment for the occurrence of ICD.

Finally, we explored the adjuvant substances in ATAA. According to the latest ICD research, the main role of adjuvant substances is to trigger chemotactic immune cells to migrate to the site of vascular lesions (10). According to the chi-square test results, in ATAA, we found that the numbers of the two most important immune cells involved in ICD [CTLs and mDCs (the strongest antigen-presenting cells (37))] were much higher than those in the control group. This finding indirectly suggested the presence of adjuvant substances in ATAA. We identified a total of 9 signaling pathways that may be related to ICD, and we discussed the most important pathway related to endothelial cell ICD here. According to the latest ICD research, the primary role of ANXA1-FPR1 in tumor cells is to direct APCs to dying cells (38). ANXA1-FPR1 most likely plays the same role in ATAA. In our study, a large amount of APC infiltration was observed in ATAA samples, and this result was also consistent with many current literature reports (4).

CXCL12 interacts with glycosaminoglycans on endothelial cells before it can be stably presented to leukocytes (39, 40). It mainly attracts NK cells and T-lymphocytes (41). In cardiovascular disease, it has been shown that CXCL12 is proatherogenic in the development and progression of atherosclerosis (41, 42). Atherosclerosis is primarily responsible for ATAA in elderly individuals (2). The mean age of ATAA patients in this study was 67.6 ± 8.1 years, which was also in line with other reports in the literature. Stéphanie Michineau et al. showed that CXCL12/CXCR4 axis is upregulated in human and mouse AAAs (abdominal aortic aneurysms), and the CXCR4 gene knockout can inhibit the expansion of abdominal aortic aneurysm through anti-inflammatory effect (43). This finding suggests that CXCL12/CXCR4 may play an important role in the formation of ATAA.

In the **Supplementary Figure 3K**, the ACKR1 gene was obviously expressed in endothelial cells. ACKR1 (typical chemokine receptor 1), also known as DARC, binds to more than 20 different inflammatory chemokines, mainly CC and CXC subfamilies (44). In previous studies, the ACKR1 gene was found to be mainly expressed on the surface of red blood cells and to a lesser extent in vascular endothelial cells and adipocytes (45). Mice with global DARC knockout had a significantly lower probability of developing atherosclerosis than wild-type mice (46). Many African Americans carry mutations in the gene encoding this receptor, resulting in a loss of its expression. This mutation resulted in a lower incidence of coronary heart disease in African Americans than in Caucasians (47). It has also been reported that ACKR1 deficiency can decrease T-cell numbers in the aorta (44). This finding suggests that AKR1 deficiency has the potential to block adaptive immunity to ICD in ATAA. In cardiovascular diseases, the protein expressed by ACKR1 gene may destroy the barrier function of endothelial cells by promoting the aggregation and transfer of endothelial cells, and promote circulating leukocytes to enter the vascular intima at the early stage of atherosclerosis (48). In skin diseases, it has been found that the interaction CXCL8/ACKR1 between macrophages and endothelial cells is enhanced, and the purpose of this interaction is to recruit immune cells to inflammatory sites in order to fight the infection (49). In the latest published article, ACKR1+ ECs (Endothelial cells) highly engaged in leukocyte recruitment into orbital connective tissue (OCT) in (thyroid-associated ophthalmopathy) TAO and that the recruitment process may be influenced by the interaction of CXCL8/ACKR1 (50). Overall, CXCL8/ACKR1 axis has reported its recruitment effect on immune cells in various diseases. This axis may also play an important role in the occurrence and development of ATAA.

As well as its function in regulating CCL2 and CCL5 activity, ACKR1 is also reported to be involved in translocating these chemokines across the endothelial barrier after it binds to them (51, 52). In the Hashimoto’s thyroiditis (HT), one study has proved that CCL5/ACKR1 axis facilitated the trans-endothelial migration of lymphocytes (53). According to our studies, the ACKR1 gene of endothelial cells in the aorta could not only promote T-cell infiltration through the CCL5 ligand but also promote myeloid cells infiltration through the CXCL8 ligand. ACKR1 could become a target gene for ATAA drug therapy in the future.

Our study has confirmed that ICD was present in ATAA and played an important role in the development of ATAA. And we used CellChat to screen three important ligand-receptor pairs (CXCL8-ACKR1, CCL5-ACKR1, CXCL12-CXCR4), and obtained two special genes expressed by endothelial cells, namely ACKR1 and CXCL12.

## 5 Conclusion

ICD is present in ATAA and plays an important role in the development of ATAA. The target cells of ICD are mainly endothelial cells, which communicate with chemotactic T cells and mature dendritic cells mainly through CXCL12-CXCR4 and myeloid cells through ANXA1-FPR1. The ACKR1 gene expressed by endothelial cells promotes the development of ATAA through the CCL5 ligand expressed by T cells and the CXCL8 ligand expressed by myeloid cells. The CXCL12 gene expressed by endothelial cells promotes the development of ATAA through the CXCR4 receptor expressed by T cells and mature dendritic cells. Overall, ACKR1 and CXCL12 may become target genes for ATAA drug therapy in the future.

## Data availability statement

Publicly available datasets were analyzed in this study. This data can be found here: https://www.ncbi.nlm.nih.gov/geo/query/acc.cgi?acc=GSE155468.

## Ethics statement

The data for this study were obtained from public databases and no additional ethical approval was required.

## Author contributions

The manuscript has been read and approved by all authors. DJ, conceptualization, funding acquisition, project administration, supervision, writing – review and editing, linguistic editing, and proofreading. XL and PZ, review and editing. ZT, data curation, formal analysis, writing – original draft, visualization, software, methodology, validation, writing – review and editing. All authors contributed to the article and approved the submitted version.
