# Supplementary figures and images for: Analysis of immunogenic cell death in ascending thoracic aortic aneurysms based on single-cell sequencing data
Source: Front Immunol. 2023 May 3;14:1087978. doi: 10.3389/fimmu.2023.1087978 (PMC10191229; doi:10.3389/fimmu.2023.1087978)

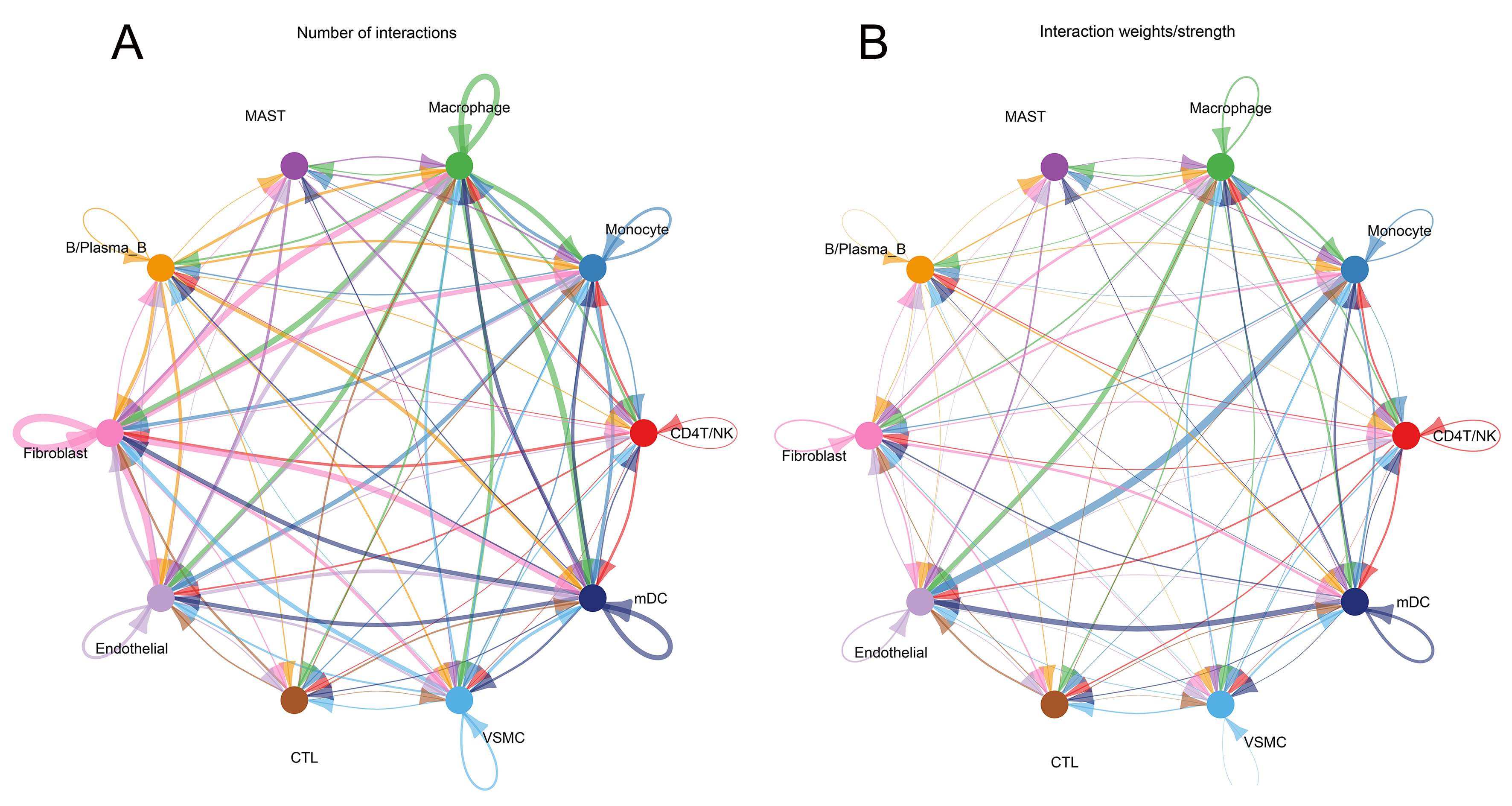

Supplement: Supplementary file 1 [file Image_1.tif]

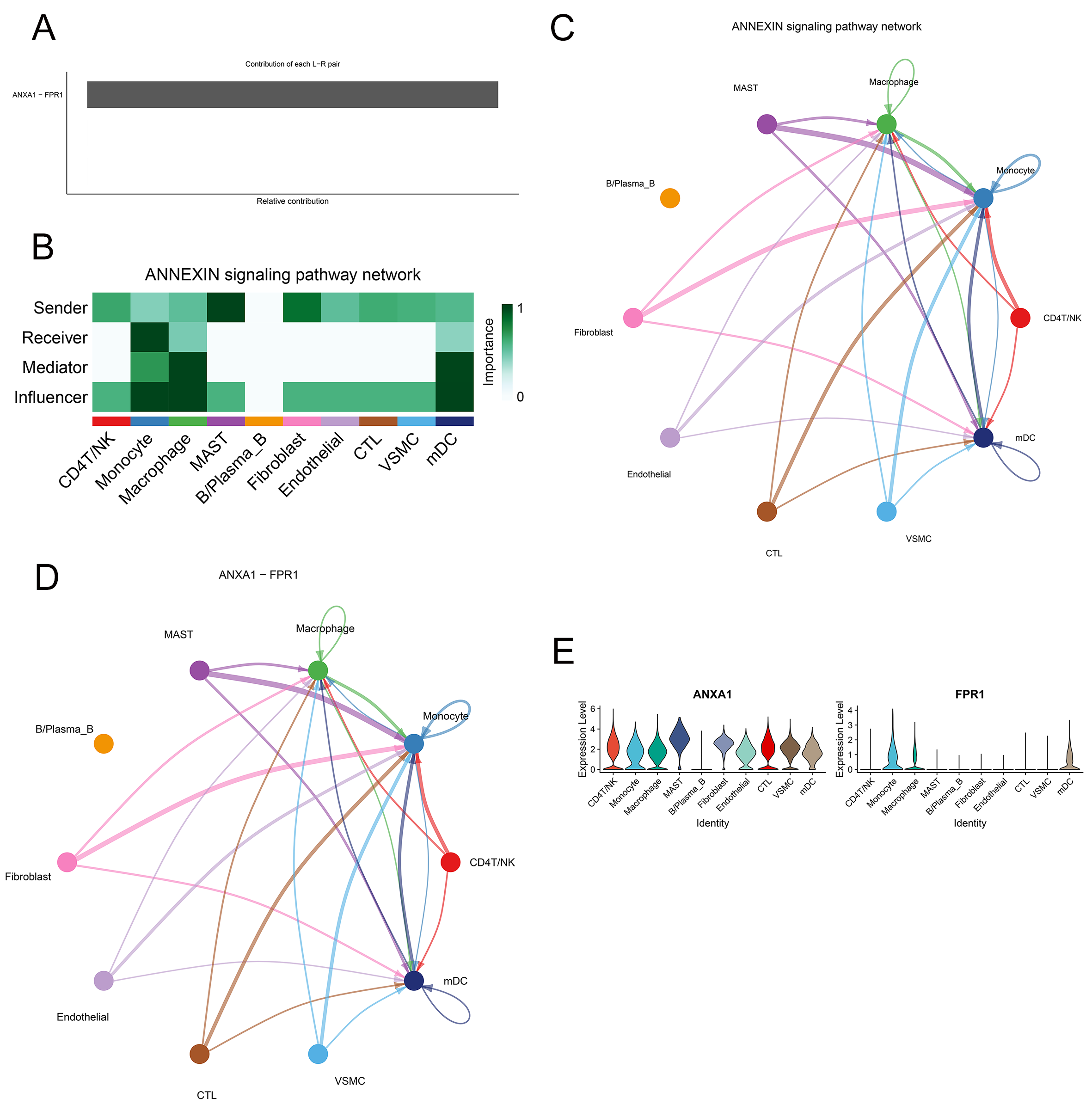

Supplement: Supplementary file 2 [file Image_2.tif]

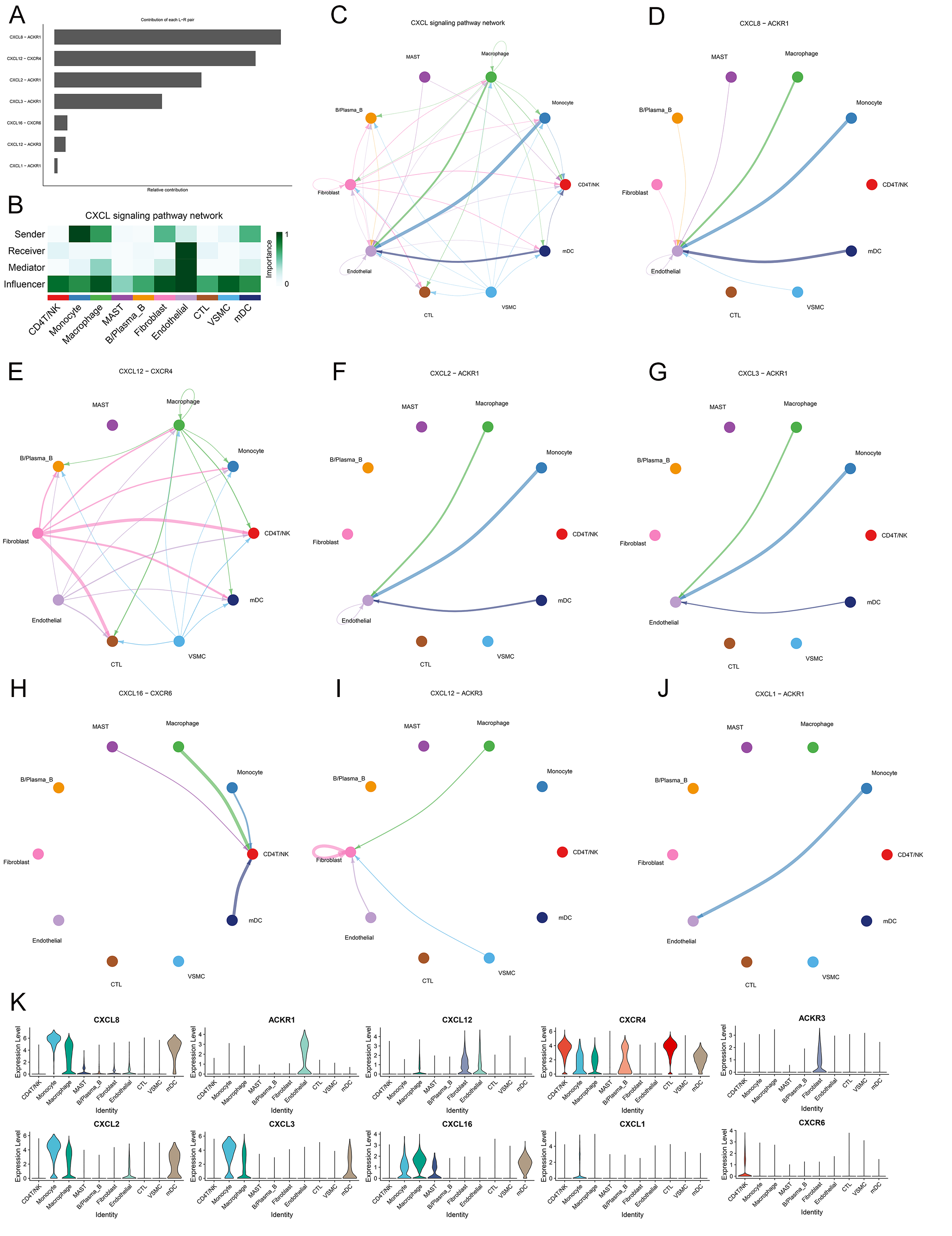

Supplement: Supplementary file 3 [file Image_3.tif]

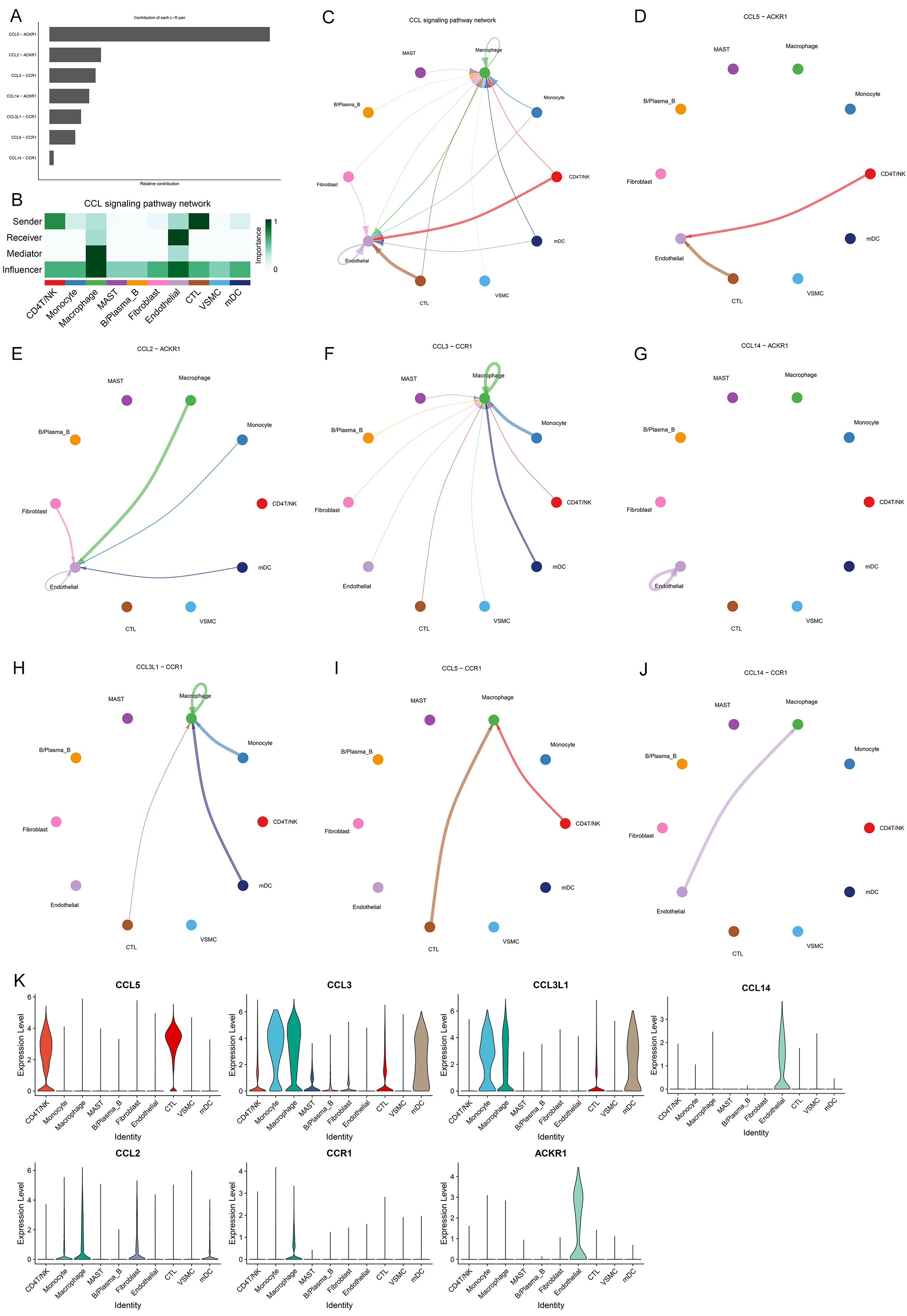

Supplement: Supplementary file 4 [file Image_4.tif]

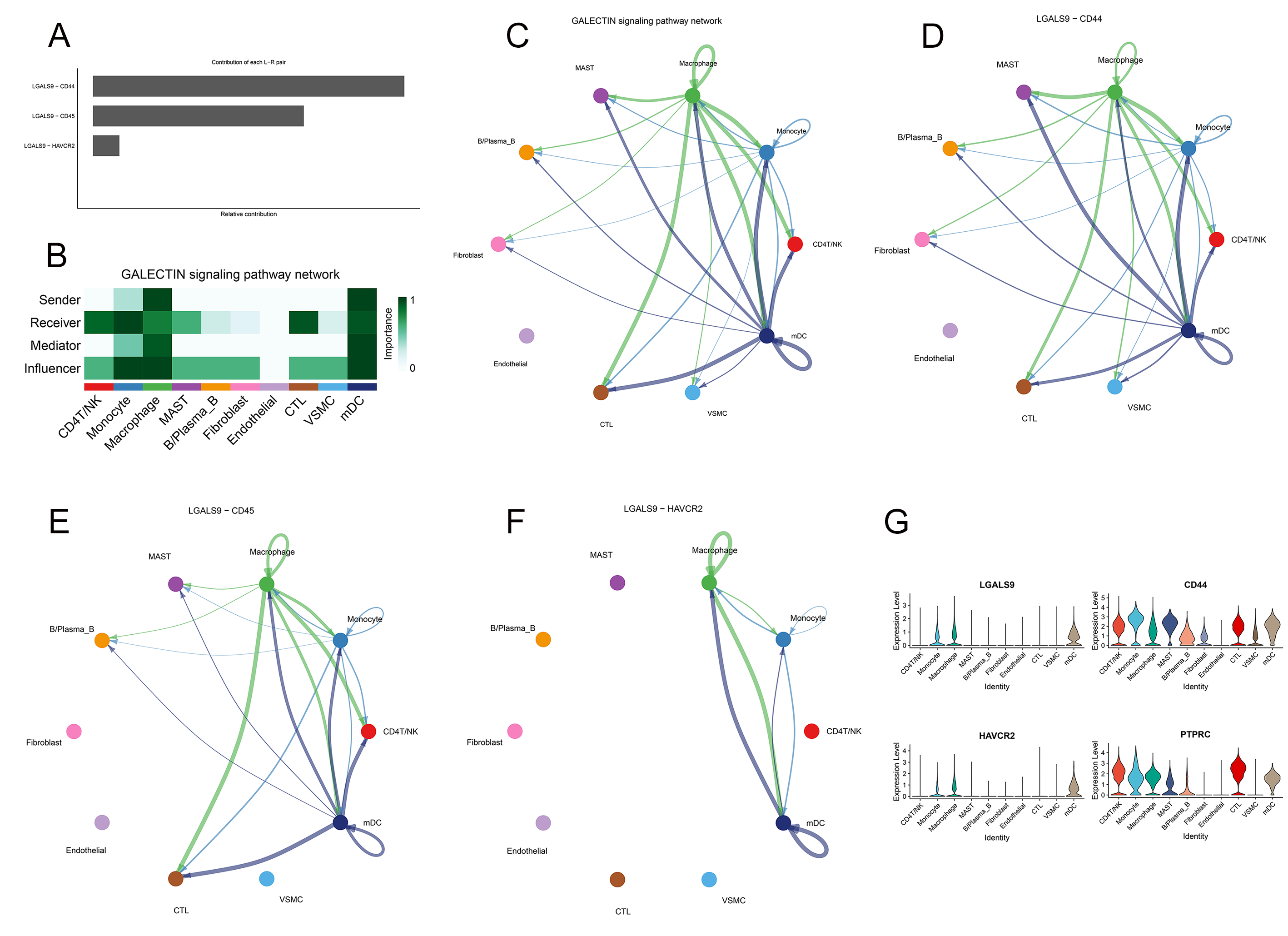

Supplement: Supplementary file 5 [file Image_5.tif]

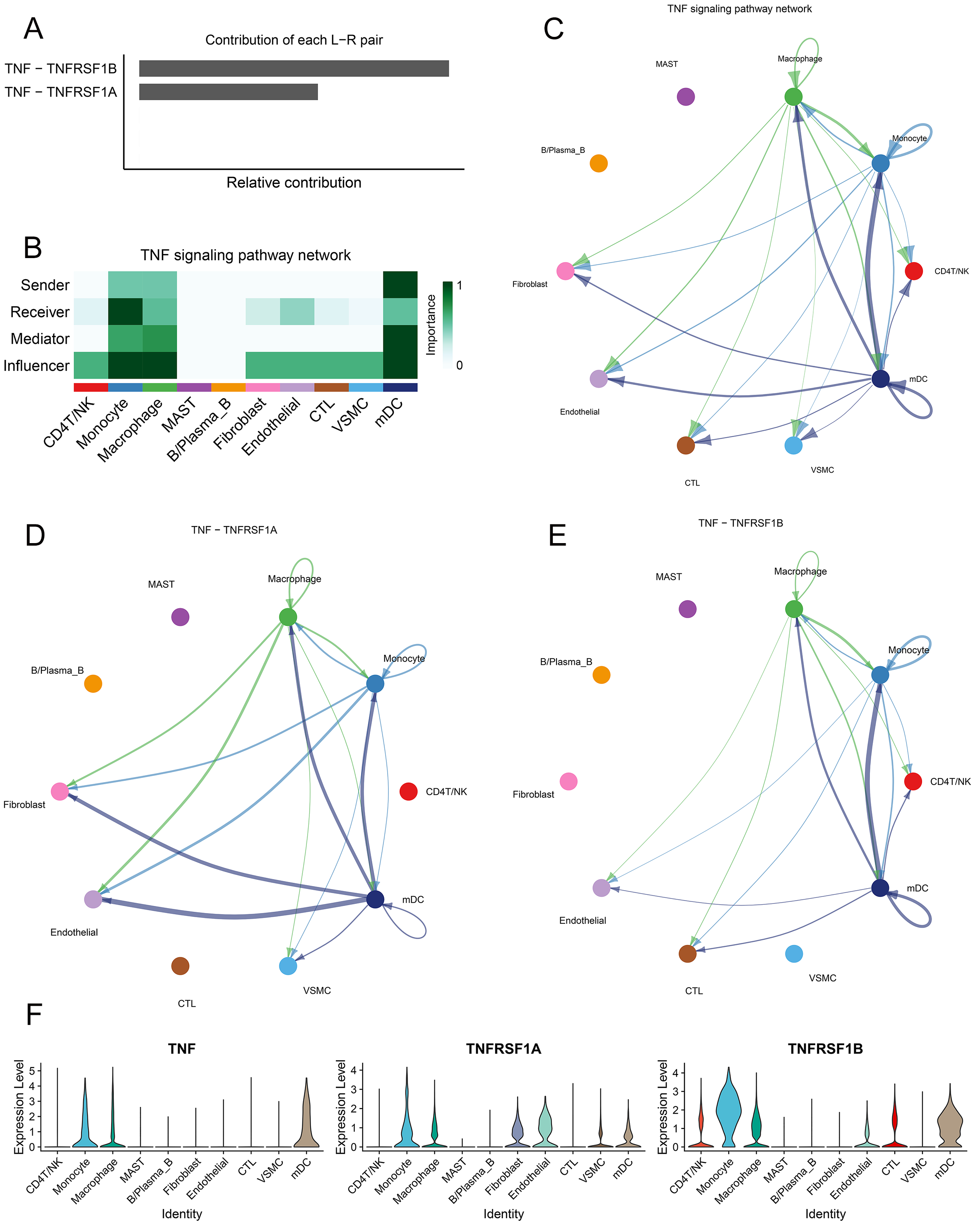

Supplement: Supplementary file 6 [file Image_6.tif]

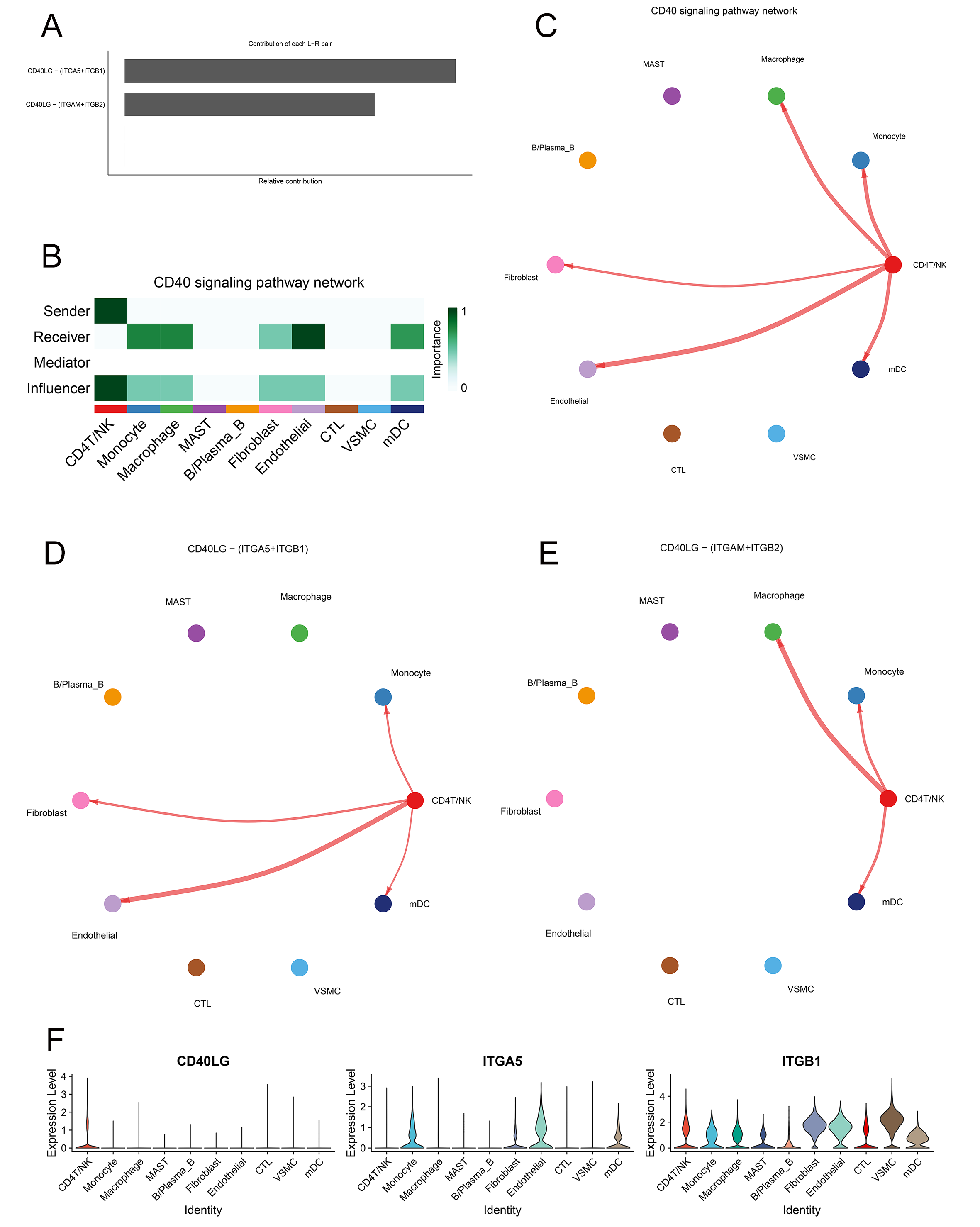

Supplement: Supplementary file 7 [file Image_7.tif]

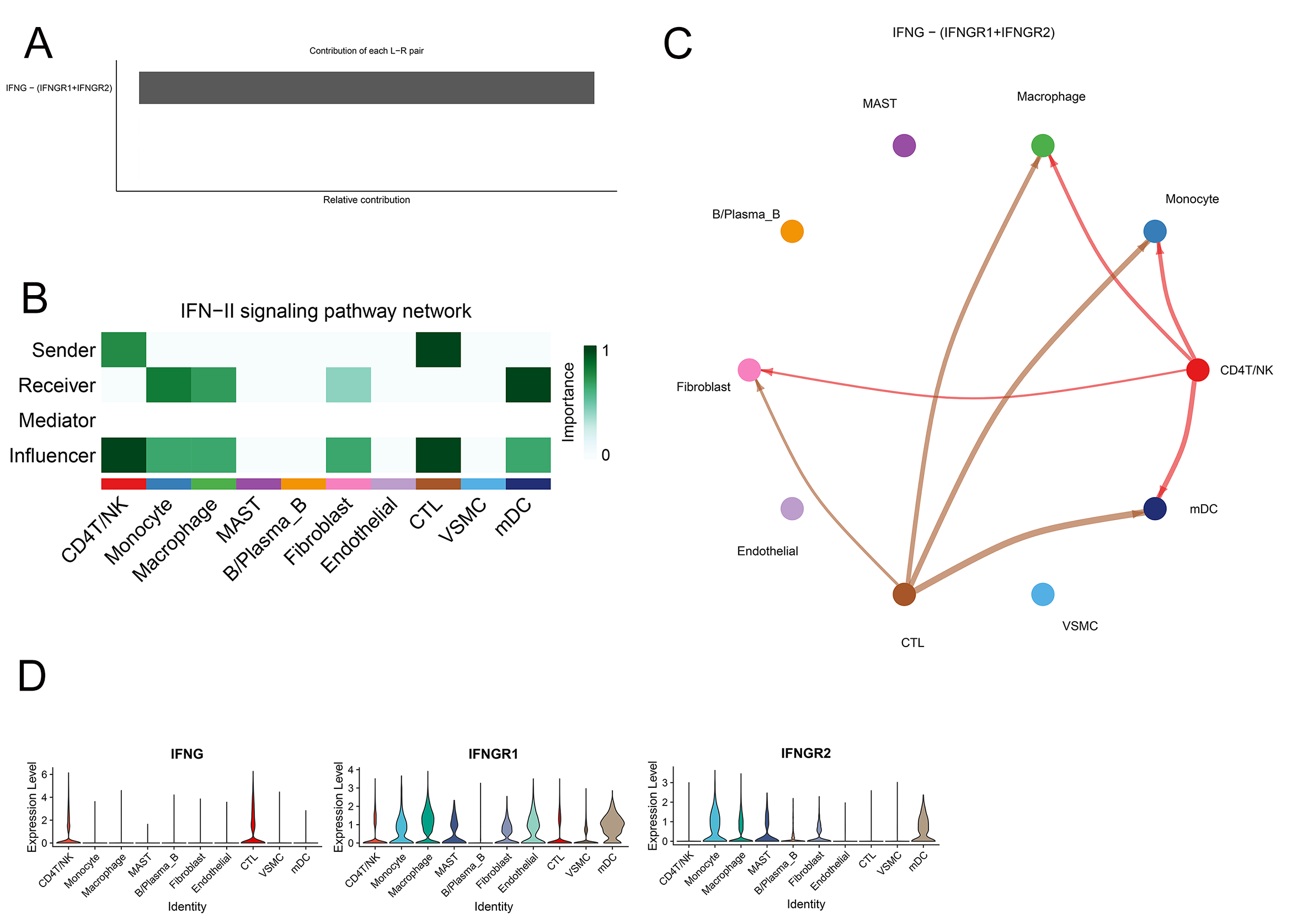

Supplement: Supplementary file 8 [file Image_8.tif]

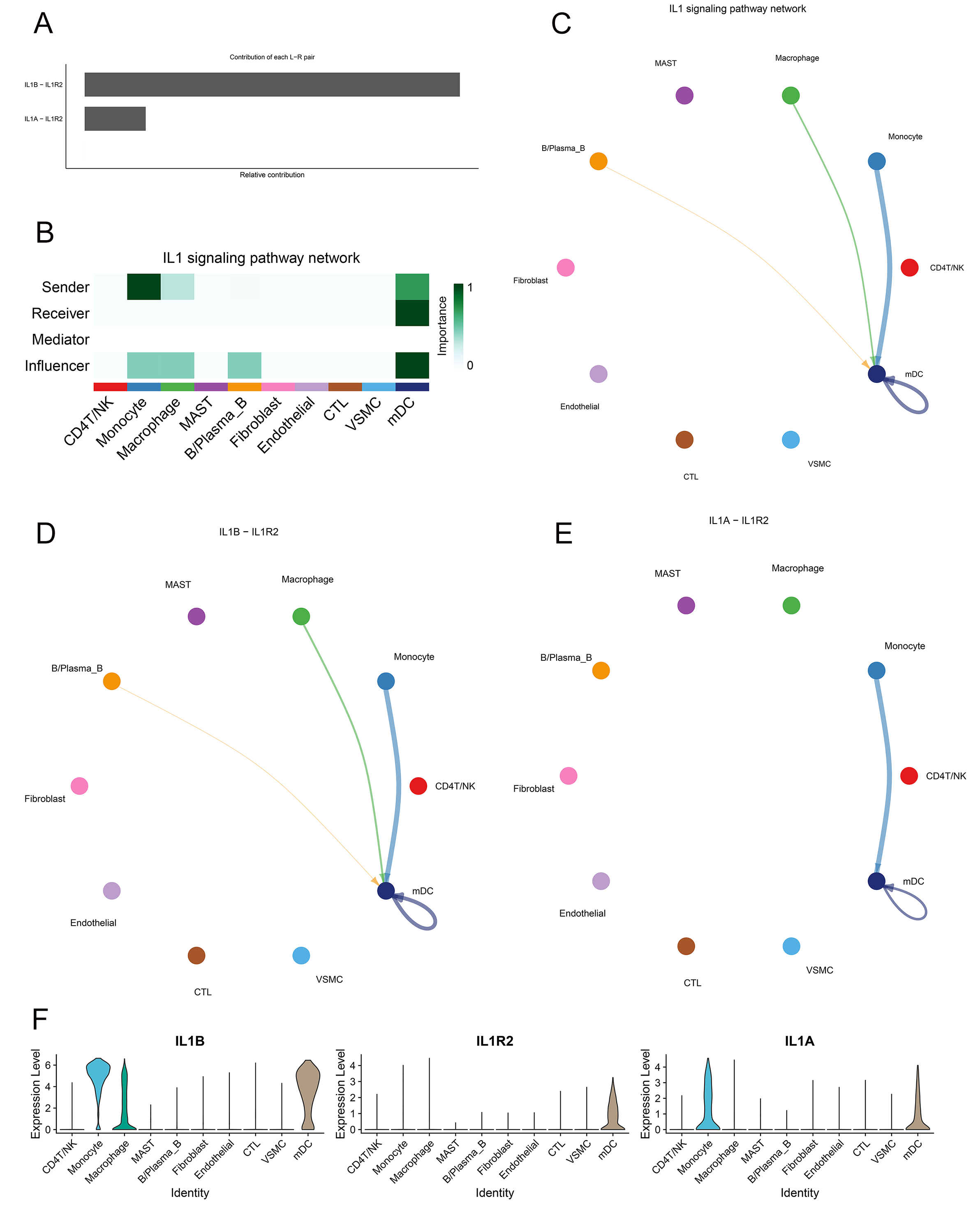

Supplement: Supplementary file 9 [file Image_9.tif]

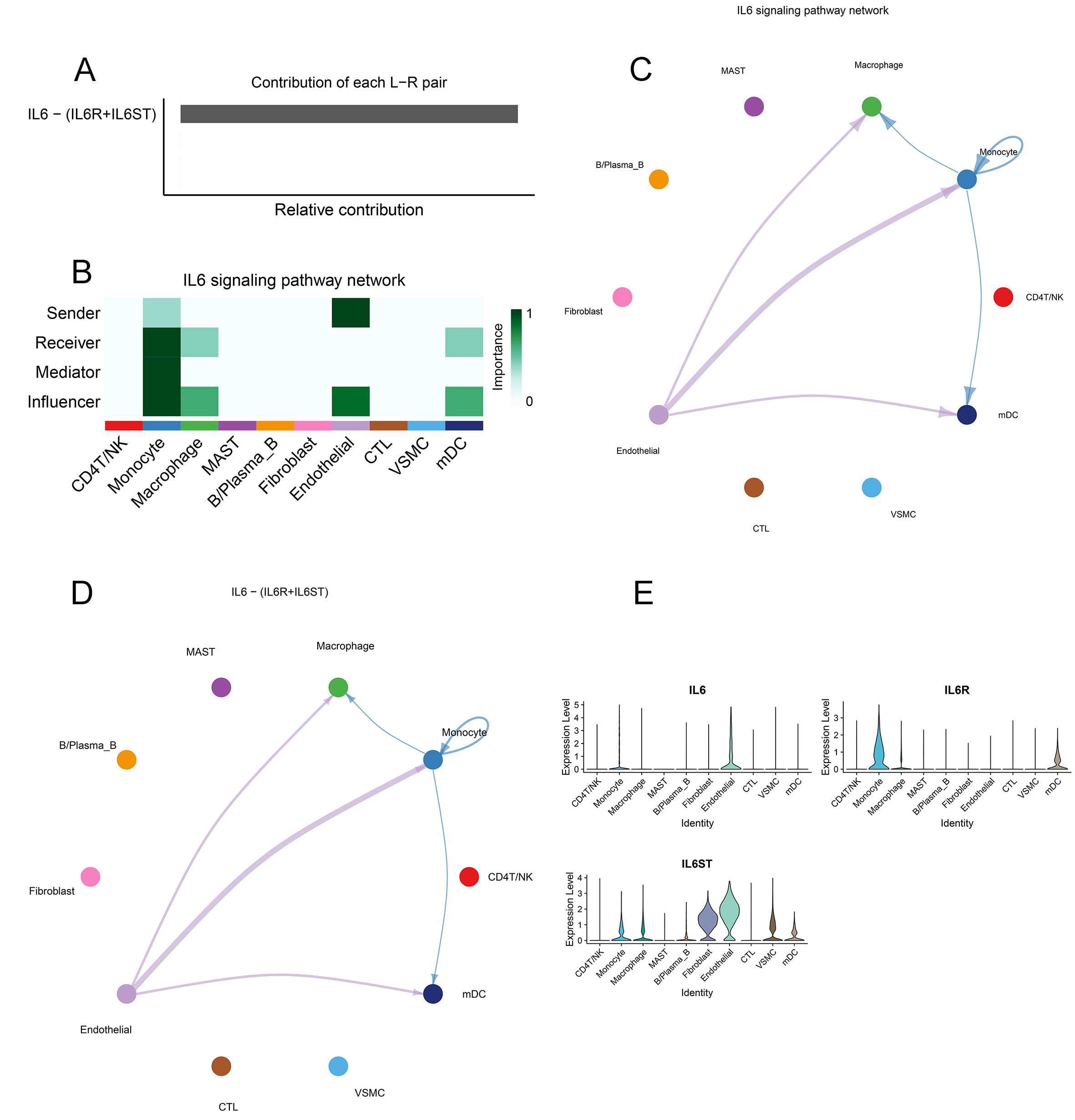

Supplement: Supplementary file 10 [file Image_10.tif]
